# Characterisation of diabetic ketoacidosis in children and adolescents with type 1 diabetes: a regional hospital study

**DOI:** 10.1186/s12887-025-05824-0

**Published:** 2025-06-07

**Authors:** Sophy Korula, Sheikh Arif Maqbool Kozgar, Lloyd Bwanaisa

**Affiliations:** 1 Paediatric Consultant and Paediatric endocrinologist, Department of Paediatrics, Latrobe Regional Health; Adjunct Lecturer and CAPS Tutor, Monash School of Rural Health, Traralgon, VIC 3844 Australia; 2 Paediatric Consultant, Department of Paediatrics and Director of Clinical Training, Latrobe Regional Health; Adjunct Senior Lecturer, Monash School of Rural Health, Traralgon, VIC 3844 Australia; 3 Paediatric Consultant and Clinical Lead Paediatrics, Department of Paediatrics, Latrobe Regional Health, Traralgon, VIC 3844 Australia

**Keywords:** DKA, Diabetic ketoacidosis, Regional hospital, T1D, Insulin, Paediatric

## Abstract

**Aim:**

To characterise the clinical and biochemical parameters of children (0–16 years) who presented with Diabetic Ketoacidosis (DKA) at a regional hospital in Australia.

**Methods:**

A retrospective observational study was conducted following the approval of the Ethics Committee. Data from 2018 to 2022 were collected from medical records, with a focus on patient treatment and follow-up.

**Results:**

A total of 72 type 1 diabetes (T1D) patients with 30 DKA presentations were identified. The mean age at DKA presentation was 11.9 +/- 3.2 years, with 42.1% having new-onset T1D. An equal number of patients presented with mild (50%) and moderate to severe DKA. Of these, 24 presentations were managed with insulin infusion, and 6 (20%) were managed with subcutaneous insulin. Following a mean ED stay of 7.93 +/- 4.8 h, 14 patients (93.3%) were transferred to the CCU or ward, and 2 were transferred to a tertiary centre. The mean HbA1c was 12.55 +/- 2.1%, with a mean recovery time of 10.4 h for pH and 6.4 h for bicarbonate. Minor complications occurred in 10% of patients (all on insulin infusion). All patients were discharged in stable condition after 2.15 +/- 1.3 days. The follow-up rate was 72.2% (13/18), with a mean HbA1c of 8.32 +/- 1.8%.

**Conclusion:**

Regional hospitals witness a high frequency of children with T1D presenting with DKA as their first presentation. Targeting bicarbonate levels for acidosis correction could help facilitate an earlier transition to subcutaneous insulin and needs due consideration. This study substantiates the use of upfront subcutaneous insulin for mild to moderate DKA with good outcomes. Follow-up care remains a crucial gap that necessitates strengthening regional diabetes management teams.

**Clinical trial number:**

Not applicable.

**Supplementary Information:**

The online version contains supplementary material available at 10.1186/s12887-025-05824-0.

## Highlights

**What is already known about this topic?**

1. Regional areas in Australia demonstrate 1.5 times higher risk of Diabetic ketoacidosis (DKA) as the first manifestation of type 1 diabetes (T1D) as compared to the cities.

2. Although the overall incidence of T1D in Australia has remained stable in recent years, the highest increase has been in regional areas.

3. DKA is conventionally managed with insulin infusion, which is labour-intensive and may lead to complications with prolonged use.

4. The 2022 International Society of Paediatric and adolescent diabetes (ISPAD) guidelines propose subcutaneous insulin for managing mild and moderate DKA in otherwise well children, but empirical data on its application, particularly in regional areas, are limited.

**What does this study add?**

1. This study confirms the increased risk of DKA as the first presentation of T1D in this regional centre.

2. Most DKA patients presenting to our regional hospital are managed locally.

3. Emphasis on bicarbonate recovery as a target for acidosis correction in the guidelines could facilitate an earlier transition to SC insulin for those initiated on an insulin infusion, potentially reducing the intensive management demand at regional centres.

4. This study establishes the safe use of upfront SC insulin in managing a specific subset of paediatric DKA patients.

**Supplementary Information:**

The online version contains supplementary material available at 10.1186/s12887-025-05824-0.

## Introduction

“Diabetes mellitus” is a complex metabolic disorder characterised by chronic hyperglycaemia due to defects in insulin secretion, insulin action, or both. The absence of insulin production or secretion is typically referred to as type 1 diabetes (T1D) [1]. The global incidence of T1D is reported to be 15 per 100,000 individuals [2], with predictive modelling indicating a rapid increase in these estimates over the next two decades [3]. In 2016, the Australian Institute of Health and Welfare (AIHW) reported an annual incidence of type 1 diabetes of 10–13 cases per 100,000 people in Australia [4], which aligns with the worldwide incidence figures. While the incidence rates of T1D in Australia have remained stable between 2020 and 2021, notably, the highest rates are observed in inner regional areas [5].

Children with T1D typically present with weight loss, polydipsia and polyuria [1]. They may also present in diabetic ketoacidosis (DKA) at onset, a life-threatening manifestation of insulin deficiency with features of hyperglycaemia, acidosis and ketones in blood and urine. In countries with a high incidence of T1D, greater familiarity among medical practitioners with the condition results in a lower occurrence of DKA at the time of diagnosis [6]. Routine childhood illnesses and missed insulin doses also result in DKA among patients known to have T1D. DKA is usually managed with fluids and insulin infusion with frequent monitoring of acid‒base and electrolytes. Cerebral oedema, a rare complication, primarily occurs in the early phase of DKA management and affects approximately 0.5% of cases [6]. This complication can lead to neurological consequences [7] and has a mortality rate as high as 40% [8]. Delayed diagnosis is identified as a major risk factor associated with mortality [9]. Therefore, prevention and timely identification of DKA and its appropriate management are crucial. However, this process is labour-intensive and can be particularly challenging in regional settings where human and infrastructural resources are often limited.

In Australia, the incidence of DKA as the initial presentation of T1D is 37.7% [10] and 31.8% [11] in major cities. In contrast, regional areas in Australia present a 1.5-fold greater risk for DKA at diagnosis, as highlighted by the AIHW report [4]. A recent study from a regional centre in the state of Queensland reported that the average incidence of DKA at the first presentation of T1D was 48.10%, with another study reporting 50%, further substantiating the increased rates observed in regional settings [12, 13]. Notably, the frequency of DKA was also significantly greater during the period of COVID-19 pandemic restrictions than during prepandemic conditions (73% vs. 26%; *P* < 0.007) [14]. Studies from regional Queensland noted that age, gender, rurality or indigenous status did not change the risk of DKA [12, 13]. However, a recent study on diabetes-related presentations to emergency departments revealed a 68% greater risk of children presenting with DKA in regional Victoria than in metropolitan areas [15]. These findings underscore the need for further research into DKA presentations in children and youth from regional areas.

There is currently no published literature characterising this life-threatening emergency in regional Victoria. According to the AIHW 2020 report, Victoria ranks as the third highest state in Australia for T1D incidence. This study was undertaken as an initial step to address the need to examine DKA presentations at a regional hospital in Victoria to enhance our understanding and improve community referral services for better childhood T1D care.

## Methods

### Ethics

This observational study was conducted with the approval of the Human Research Ethics Committee at Latrobe Regional Health (LRH), project no. 2023-47 QA.

### Setting

LRH is a regional health service in Victoria, Australia that caters to the needs of the Latrobe Valley, covering an area of 1,426 square kilometres and a population of around 78,000. Children 0–14 years contribute to approximately 20% of the population. This healthcare service is a secondary care academic institution in a rural setting.

### Study design and data collection

This is an observational study for which data was obtained from the medical records of all children and adolescents (aged 0–16 years) admitted to LRH over five years, from 1st Jan 2018 to 31st Dec 2022, who had T1D or DKA (codes E1011 and E109). Basic demographic data, type of DKA, treatment details and place of treatment within the hospital were obtained from Electronic Medical Records (EMR or Sunrise™, Altera Digital Health). Outpatient clinic software Genie^®^ (Practice Management Software, Magentus) was used to obtain follow-up details, including type of insulin, duration of follow-up and HbA1c. In 2020, the hospital transitioned to a fully electronic record system, so the data collection included written charts and electronic records. The data were coded and entered into a password-protected Microsoft Excel spreadsheet in a confidential, de-identified manner by a single author (SK) with access restricted solely to the investigators. No personal computers or data devices were used to collect, transfer or store data.

### Definitions

Patients were classified into diabetic ketosis, mild, moderate, and severe DKA using the ISPAD guideline definitions [1] based on the initial blood gas measurement (venous, arterial or capillary). Capillary glucose, as noted on the glucometer, and blood ketones, as noted using ketone strips, were included. The study analysis focused on patients who presented with DKA. The point of pH recovery was determined as when the pH level exceeded 7.30, and bicarbonate recovery as the time at which bicarbonate levels surpassed 15 mmol/l by any one of venous, arterial or capillary gas measurements.

### Statistical analyses

**Fig. 1:**
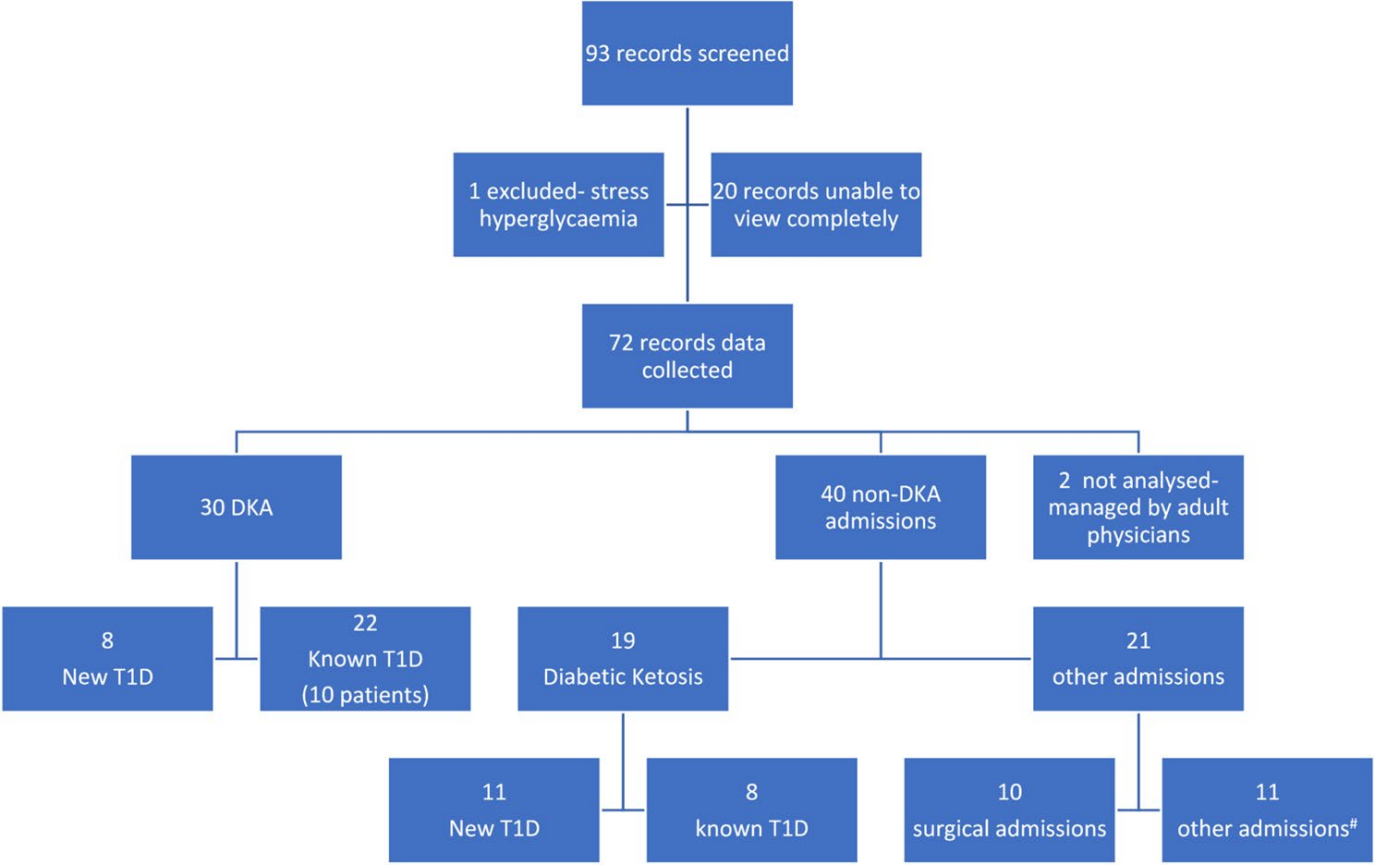
Study flow diagram # Total of 11 − 1 for mental health concerns, 4 for re-education due to poor control, 2 in preterm labour, 2 with hypoglycaemia, 2 for evaluation of musculoskeletal pain

Statistical analyses- Simple descriptive statistics using mean and standard deviation, and proportions were used for the analysis. No tests of significance were employed. Selection bias was addressed by ensuring only those with eligible hospital codes were included. Data loss due to the absence of paper charts in 20/93 screened is a potential bias (Fig. 1).

The manuscript was prepared per the STROBE guidelines for reporting observational studies. The main aim of the study was to characterise the baseline clinical and biochemical parameters of DKA patients.

The specific objectives to be studied were defined as follows:

1. Calculation of the mean duration for recovery of pH and bicarbonate levels.
2. Assessment of in-hospital disposition (ward/CCU/ED), the mean duration of hospital stays and the occurrence of complications.
3. Follow-up data analysis.
4. Description of the cohort of patients managed with subcutaneous insulin from the outset.

## Results

Out of 93 records screened, data were collected from 72 children with T1D who presented to the emergency department and inpatient services between 2018 and 2022. Owing to the maintenance of paper records before 2020, charts for 20 patients, which were missing, could not be screened, and one patient excluded, had stress hyperglycaemia. A total of 30 DKA presentations were recorded over 5 years, comprising 16 male patients and 14 female patients. Eighteen patients accounted for the 30 DKA presentations, with 5 patients experiencing multiple admissions during this period. DKA was identified as the first manifestation of T1D in 42.1% (8/19) of patients. Figure 1 illustrates the flowchart of the study flow process.

## Baseline clinical and biochemical characteristics

The baseline characteristics of the patients are summarised in Table 1, with continuous variables expressed as mean ± standard deviation and categorical variables as numbers or percentages. The mean age of patients at DKA presentation was 11.9 ± 3.2 years. According to the ISPAD guidelines, 50% of the cases were classified as mild DKA (15/30), 26.7% as moderate DKA (8/30) and 23.3% as severe DKA (7/30). At the time of presentation, the mean pH was 7.18 ± 0.09, with a mean venous bicarbonate of 12.35 ± 3.29 mmol/L. The mean venous glucose level was 28.77 ± 8.14 mmol/L, and the mean blood ketone level recorded by the glucometer was 5.5 ± 0.86 mmol/L. The mean HbA1c at DKA presentation (*n* = 11) was 12.55 ± 2.06%. Twenty-four patients received insulin infusion for a mean duration of 10.72 ± 8.99 h, while 6 patients (20%) were treated with subcutaneous insulin. Two patients at presentation received continuous subcutaneous insulin infusion (CSII), while the remaining patients were either on or initiated multiple daily subcutaneous insulin injections with a basal-bolus regimen. All patients belonged to the most disadvantaged category as per the IRSAD classification.

**Table 1:** Baseline patient profile (*n* = 30)

Parameter	Value
Total Patients with DKA	18
Patients with Multiple DKA Admissions	5
Frequency of DKA presentations	
2018 2019 2020 2021 2022	5 4 4 4 13
Age at DKA presentation (years)	11.9 ± 3.2
Age at T1D diagnosis (years)	8.9 ± 4.4
Gender	
Males Females	16 14
DKA Severity Distribution	
Mild DKA Moderate DKA Severe DKA	50% (15/30) 26.7% (8/30) 23.3% (7/30)
Biochemical parameters at presentation	
pH Bicarbonate (mmol/L) Glucose (mmol/L) Blood Ketone (mmol/L) HbA1c (*n* = 11)	7.18 ± 0.09 12.58 ± 3.16 28.77 ± 8.14 5.5 ± 0.86 12.55 ± 2.06%

### Evaluation of patient outcomes

The results of the analysis of recovery times and other patient outcomes are summarised in Table 2, with continuous variables expressed as mean ± standard deviation and categorical variables as numbers or percentages. Among the 18 patients who presented, 2 were on CSII, and 16 were on multiple daily SC insulin. The same insulin delivery regime with adjusted doses was continued after DKA management.

### Mean recovery time for pH and bicarbonate levels

The mean duration for pH recovery was 10.36 ± 7.79 h, whereas bicarbonate recovery occurred in 6.41 ± 5.44 h. In all instances, bicarbonate recovery occurred earlier than pH recovery, except for two cases where gastrointestinal illness precipitated DKA.

### In-hospital outcomes: disposition, length of stay and complications

All patients were initially managed in the Emergency Department (ED), with an average duration of ED stay of 7.93 ± 4.85 h. Fourteen patients were subsequently transferred to the critical care unit, and another 14 were moved to the paediatric ward in the hospital. Two patients were directly transferred to a tertiary care centre from the ED after consultation with retrieval services. One patient who had recently undergone surgery for a complex heart condition was transferred from the CCU to the tertiary centre due to concerns of coagulopathy following recovery from DKA. The average length of hospital stay was 2.15 ± 1.32 days, with a mean of 3.7 days in the insulin infusion group and 1.7 days in the SC insulin group. Three patients required active intervention for complications: one patient with hyperkalaemia (potassium 6.4 mmol/L) had potassium supplementation decreased from 60 to 30 ml/L in the fluids; another patient was noted to have both hypokalaemia and hypoglycaemia. The patient received oral potassium supplementation for hypokalaemia (potassium 3.1 mmol/L) and a dextrose bolus along with a temporary suspension of the insulin infusion for an hour for a glucose level of 2.1 mmol/L. Additionally, a third patient with hypophosphatemia was given oral phosphate supplementation. All 28 patients managed at LRH were discharged in a stable condition.

### Post-hospitalisation follow-up: percentage of patients with more than one year of follow-up and average follow-up duration

At the time of data entry, 13 out of 18 patients were followed up at the hospital clinic for a mean duration of 1.65 ± 1.62 years following their initial presentation. The mean HbA1c at the last follow-up was 8.32 ± 1.80%, with a mean total daily insulin dose of 0.96 U/kg/day. Two patients were transferred to other local services for ongoing follow-up, and one was transitioned to adult endocrine services at LRH. Additionally, two patients did not return to the outpatient clinic after their DKA was managed and were thus considered lost to follow-up.

**Table 2:** Summary of the outcome measures

Outcome Measures	Value
Number of patients initiated on insulin Infusion	24
Number of patients initiated on subcutaneous insulin	6
Duration of insulin infusion (hours)	10.72 ± 8.99
Time to pH Recovery (hours)	10.67 ± 7.85
Time to Bicarbonate Recovery (hours)	6.79 5.5 ± 4
ED Stay Duration (hours)	7.93 ± 4.80
Transfers post-ED stay	
Critical Care Unit Paediatric Ward Tertiary Care Centre	14 patients 14 patients 2 patients
Length of Hospital Stay (days) Insulin Infusion Group (24) SC Insulin Group (6)	2.18 ± 1.3 2.3 1.7
Complications Needing Intervention	3 patients
Discharge Condition	All 28 patients are stable.
Follow-up post-hospitalisation at our centre	13/18 patients
Duration of Follow-Up (years)	1.65 ± 1.62
HbA1c at Last Follow-Up (%)	8.32 ± 1.80
Insulin Dose at Follow-Up (U/kg/day)	0.96
Transfers to Other Services	3 patients (2 local, 1 adult endocrine)
Loss to Follow-Up	2 patients

### Overview of patients managed with subcutaneous insulin from the outset

A total of 20% (6/30) of patients received subcutaneous insulin from the beginning of their treatment. The characteristics of this cohort are summarised in Table 3. Most of these patients presented with mild DKA (5/6), whereas one patient presented with severe DKA. The mean time to pH recovery was 2.67 +/- 3.1 h, and that to bicarbonate recovery was 2.75 ± 2.13 h. The mean duration of ED stay was 9.67 ± 5.9 h, whereas the mean in-hospital stay was 1.67 ± 1 days.

**Table 3:** Characteristics of children and adolescents with DKA managed with subcutaneous insulin

Case	Age	Gender	DKA Severity	pH at Presentation	Bicarbonate at Presentation	Time to pH Recovery	Time to Bicarbonate Recovery	ED Stay Duration (hours)	Length of Hospital Stay (days)
1	8	F	Mild	7.25	16.3	3	0	8	1
2	15	M	Mild	7.29	16	1	0	4	1
3	14	M	Mild	7.28	17	0	2.5	9	3
4	12	F	Mild	7.202	13	4	4	21 (delay due to bed shortage)	1
5	11	F	Mild	7.3	14.3	0	1	7	3
6	13	F	Severe	7.08	11	8	6	9	1

## Discussion

T1D is a chronic condition that places a significant burden on both families and the health care system. When a child is diagnosed with DKA, initial management is particularly labour-intensive and requires intravenous insulin infusion, hourly glucose and ketone checks and frequent venous gas analyses to monitor the associated acidosis.

To our knowledge, this study represents the first review of this life-threatening emergency from a regional perspective in Victoria, Australia. We observed a high incidence of DKA among newly diagnosed T1D patients (42.1%). This figure exceeds the incidences reported in previous studies from urban Australia, which reported rates of 31.8% and 37.7% [9, 10]. A more recent publication by the Australian Data Network (ADDN) examining a large cohort reported that 33.2% of patients presented initially with DKA [16]. In contrast, international data from a large multicentre study involving centres from developed countries across 3 continents revealed a lower rate of 29.9% [17]. This presents a notable contrast to figures in Australia, particularly from regional Queensland figures of 48.1% and 50% [12, 13]. While our study revealed a lower DKA incidence than in regional Queensland, it aligns with the broader trend of increased rates of DKA as the first presentation of T1D in regional centres across Australia. This may be attributed to delayed referrals and a lower awareness of the condition among the regional population and the local medical community. Notably, increased education for health professionals led to a significant reduction in incidence from 54.9 to 25%, as noted in a study from regional Queensland [12]. Moreover, socioeconomic indices classify many regional areas as disadvantaged. A recent study analysed 10 years of data (2008–2018) on DKA presentations via an ED database in Victoria. This study revealed a 68% increase in DKA presentations in rural ED’s compared with metropolitan areas, despite an overall decline in DKA presentations across ED’s during this period [15]. Data from the ADDN study also indicated that individuals residing in lower SES postcodes were significantly associated with higher HbA1c levels at follow-up [16]. According to the IRSAD, the entire Latrobe Valley, which is serviced by our hospital, is classified within the most disadvantaged category in Victoria [18]. This social determinant could also contribute to the higher incidence of DKA in this study.

Most of the patients were treated at our regional hospital, with only 6.7% (2 out of 30) requiring transfer to a referral centre. These data align with those of a Victorian ED-based study, which revealed that 10% of the patients were transferred out during 2017–2018 [15]. This highlights a crucial opportunity, as strengthening diabetes care in regional centres could lead to improved emergency management locally and better utilisation of the existing infrastructure in regional hospitals.

The subcutaneous insulin regimen can effectively manage paediatric DKA [19], particularly in regional hospitals. However, the medical team must make informed decisions regarding its applicability and patient selection. The new ISPAD guidelines, published in 2022, support the use of subcutaneous insulin for treating uncomplicated mild to moderate DKA [20]. In our study, 20% of the patients were managed with subcutaneous insulin, including one patient with severe DKA, and favourable outcomes were achieved. This evidence may encourage regional hospitals to consider SC insulin therapy for DKA, potentially reducing complications and the overall burden of care. Further research is necessary to validate these findings and to shift the perspective of the health care professionals involved, ultimately influencing their practices and the local guidelines. Notably, bicarbonate recovery in this study occurred almost 4 h earlier than did pH recovery. While most local Australian protocols mention both criteria for transition to SC therapy, they lack clarity on whether it should be pH “or” bicarbonate or both pH “and” bicarbonate recovery. This ambiguity leaves the decision to transition to SC therapy dependent on the clinical judgement of the treating paediatrician. More clarity on the same would thus be beneficial.

In this study, the average length of ED stay was 7.93 h, and the average length of in-hospital stay was 2.18 days. Estimates from the United States of America indicate that the average cost of hospital admission for DKA is 27,000 USD, whereas equivalent data from Australia are lacking [18, 21]. Thus, ensuring that protocols prioritise patient safety while minimising hospital stays can help decrease the economic burden on the healthcare system.

In our study, 18 patients accounted for 30 episodes of DKA, with 5 of these patients experiencing recurrent presentations. The primary contributing factors identified from medical records include a lack of adherence to sick day management protocols, mental health concerns and various social factors. This underscores the importance of a multidisciplinary allied health team in regional centres providing care for T1D patients. However, a comprehensive, in-depth analysis of these factors was not feasible because of the observational nature of this study.

The importance of ensuring a smooth transition of care from an inpatient setting to outpatient follow-up for all individuals diagnosed with T1D presenting with DKA cannot be overstated, as T1D is a chronic condition. Despite its significance, the current literature indicates a lack of data on the transitions from regional centres in Australia. In our study, 72.2% (13/18) of patients were followed up, with 3 transitioning to another local service for ongoing diabetes care, and 2 were lost to follow-up. Further investigation into the reasons for loss to follow-up is necessary to gain insights and address any obstacles in the future. Research suggests that presenting with DKA at diagnosis, especially severe DKA, is associated with higher HbA1c at follow-up [16, 22]. Although our incidence of DKA presentation was higher, the percentage of those with severe DKA (23.3%) was lower than the 29.3% reported in the ADDN study [16]. A more structured and longer follow-up analysis will provide more insight into the glycaemic control of children in our region.

Our study has a few limitations. This was a retrospective study, and we were unable to review the records of all the patients. Additionally, our research is based on data from a single regional centre in Victoria, which may not represent all regional centres across Australia or the world. The number of patients receiving subcutaneous insulin was limited, making it difficult to draw out significant differences compared with insulin infusion. Importantly, our study concluded in December 2022, shortly after the ISPAD guidelines, which endorsed the use of subcutaneous insulin in DKA, were published in early 2022. Data from 2023 onwards will provide further clarity on this topic.

## Conclusion

This study was designed to address the knowledge gap regarding the incidence of DKA in regional Victoria. A substantial portion of 42.1% of the new paediatric T1D patients presented with DKA to our regional centre in Australia. Notably, over 28 out of 30 (93.3%) of these patients were managed locally, and one-fifth were treated with subcutaneous insulin, achieving favourable outcomes in line with the ISPAD guideline recommendations published in 2022. The findings from this study provide a better understanding of the biochemical abnormalities and intensive management required for children with DKA during hospitalisation. We hope that this study will increase awareness in the region, potentially leading to early diagnoses and a reduction in the period prevalence of DKA associated with new-onset T1D. Additionally, we advocate for a greater emphasis on using SC insulin in DKA management. Furthermore, we call for more research on DKA in regional Australia to understand the factors contributing to the increased risk of DKA in these areas and to explore the social factors influencing adherence to insulin therapy. Strengthening health support offered by diabetes teams regionally is crucial for improving follow-up and achieving better health outcomes for individuals affected by this prevalent noncommunicable disease.

## Electronic supplementary material

Below is the link to the electronic supplementary material.

Supplementary Material 1

## Data Availability

Data spreadsheets in Microsoft excel format has been included in the supplementary files section.

## Abbreviations

DKA: Diabetic ketoacidosis
T1D: Type 1 Diabetes
ED: Emergency Department
CCU: Critical Care Unit
LRH: Latrobe Regional Health
ISPAD: International Society of Paediatric and Adolescent Diabetes
SC: Subcutaneous
AIHW: Australian Institute of Health and Welfare
IRSAD: Index of relative socioeconomic advantage and disadvantage
